# Is Diet a Determining Factor in the Induction of Gingival Inflammation by Dental Plaque? A Secondary Analysis of Clinical Studies

**DOI:** 10.3390/nu16070923

**Published:** 2024-03-22

**Authors:** Johan Peter Woelber, Valentin Bartha, Stefan Baumgartner, Christian Tennert, Ulrich Schlagenhauf, Petra Ratka-Krüger, Kirstin Vach

**Affiliations:** 1 Policlinic of Operative Dentistry, Periodontology, and Pediatric Dentistry, Medical Faculty Carl Gustav Carus, Technische Universität Dresden, Fetscherstraße 74, 01307 Dresden, Germany; 2 Center for Conservative Dentistry and Periodontology, Heidelberg University, Im Neuenheimer Feld 400, 69120 Heidelberg, Germany; valentin.bartha@med.uni-heidelberg.de; 3 Clinic of Orthodontics and Pediatric Dentistry, Center of Dental Medicine, University of Zurich, 8032 Zurich, Switzerland; sb@kfo-davos.ch; 4 Department of Restorative, Preventive and Pediatric Dentistry, School of Dental Medicine, University of Bern, Freiburgstrasse 7, 3010 Bern, Switzerland; christian.tennert@unibe.ch; 5 Department of Periodontology, University Hospital Würzburg, Pleicherwall 2, 97070 Würzburg, Germany; schlagenha_u@ukw.de; 6 Department of Operative Dentistry and Periodontology, Faculty of Medicine, University of Freiburg, 79106 Freiburg, Germany; petra.ratka-krueger@uniklinik-freiburg.de; 7 Hannover Medical School, Department of Conservative Dentistry, Periodontology and Preventive Dentistry, Carl-Neuberg-Str. 1, 30625 Hannover, Germany; vach.kirstin@mh-hannover.de; 8 Institute of Medical Biometry and Statistics, Faculty of Medicine and Medical Center, University of Freiburg, Stefan-Meier-Str. 26, 79104 Freiburg, Germany

**Keywords:** plaque, gingival inflammation, diet, nutrition, gingivitis, human

## Abstract

The aim was to determine the association between plaque and gingival inflammation reported by dietary interventions. Data of four clinical studies dealing with changed nutrition and gingival examination were reanalyzed with regard to gingival inflammation (GI), plaque (PI), and bleeding on probing (BOP). Dietary changes basically involved avoiding sugar, white flour and sweetened drinks and focusing on whole foods for 4 weeks. The control groups were to maintain their usual diet. All participants had to reduce their oral hygiene efforts. Linear regression models taking the clustering of the data due to several studies into account were applied. In total, data of 92 participants (control groups: 39, test-groups 53) were reanalyzed. While both groups showed a slight increase in dental plaque, only the test groups showed a significant decrease in inflammatory parameters: GI (mean value difference End-Baseline (Δ): −0.31 (±SD 0.36)) and BOP (Δ: −15.39% (±16.07)), both *p* < 0.001. In the control groups, there was a constant relation between PI and GI, while the experimental group showed a decreasing relationship in GI/PI (*p* = 0.016), and even an inverted relationship BOP/PI under a changed diet (*p* = 0.031). In conclusion, diet seems to be a determining factor how the gingiva reacts towards dental plaque.

## 1. Introduction

The manifestation of gingival inflammation is considered to be the main initial prerequisite for the development of periodontitis [1,2,3]. It is a highly prevalent condition in humans in industrialized countries and central aim for primary and secondary prevention of periodontitis [4,5]. With regard to the etiology of gingival inflammation, it was identified that dental plaque plays a central role in the development and resolution of gingival inflammation. A landmark-study by Löe, Theilade and Jensen [6] was one of the first studies showing that an increase in dental plaque was accompanied with an increase in gingival inflammation and that controlling plaque (by tooth brushing and interdental cleaning) resulted in a profound decrease in gingival inflammation. Since the description of this “experimental gingivitis model” the use of plaque control to counteract gingival inflammation got both proven by many studies and found its description in clinical guidelines and recommendations [3,4]. The findings of Löe et al. were even reflected in etiological models, where the “unspecific plaque hypothesis” did describe plaque itself as the central etiological factor in the etiology of gingivitis and periodontitis, respectively [7]. Due to the investigation of specific pathogens involved in the pathological process of gingival inflammation (such as the periodontal complexes described by Socransky et al. [8] this hypothesis was replaced by the “specific plaque hypothesis”. Later on, this hypothesis was extended by the “ecological plaque hypothesis” by Marsh [9], the “key stone hypothesis” by Hajishengallis [10], and the “integrated hypothesis” [11] emphasizing the environmental and inflammatory conditions for the presence of inflammophilic bacteria [12]. Due to the latter hypotheses, host-modulatory considerations were added to the classic therapeutic approach of plaque control [13]. Nonetheless, plaque control remained a core-concept in therapeutic recommendations [3,4] and plaque is still described as the “inducing” factor of gingival inflammation in central classifications [1].

While plaque control established as efficient method in daily prevention, However, it could not unequivocally be proven that an increase in plaque values was necessarily associated with an increase in gingival inflammation. Brecx et al. were one of the first researchers who reproduced the experimental gingivitis model [14]. While only one of the participants reacted in the same way like in the study by Löe et al. after 14 days of abolished oral hygiene, most of participants reacted only with mild gingival inflammation, and one participant did not develop gingivitis at all. In a further landmark study by Baumgartner et al. [15] investigating ten participants without oral hygiene for even four weeks under stone-age conditions, the known correlation between plaque accumulation and gingival inflammation was fundamentally disturbed. Even though the participants showed plaque accumulation comparable to the participants of Löe et al., the gingival index (GI) did not increase and the bleeding on probing (BOP) was even reduced by half. The study authors concluded that the principles of the experimental gingivitis model were not applicable in the absence of processed carbohydrates such as sugar. The results of this study were limited due to a missing control group and a lot of further possible influencing factors besides the diet such as physical activity, stress, or sleep. Nonetheless, the study evokes the interesting question on how wild-living mammals and early ancestors of Homo sapiens were able to cope with an accumulating biofilm in the natural absence of oral hygiene. From an evolutionary, biological, and nutritional point of view, there is evidence that cultural changes from the Hunter–Gatherer period to modern times led to a dramatic increase in risk factors (like processed diets, smoking, and chronic stress) and accordingly to an increase in the pathogenicity of the oral biofilm [16]. Based on these considerations, the participants of the original experimental gingivitis study of Löe et al. have already been exposed to the fundamental inflammation-promoting environment of the modern times, although the researches were not aware of it.

In this context, diet seems to be a profound etiological part of this increase in oral and periodontal pathologies from Hunter–Gatherer to modern times [17,18], mainly triggered by the Neolithic and Industrial Revolutions [16]. Approaches reconstructing the dietary changes from Hunter–Gatherer to modern times show a dramatic increase in processed carbohydrates (such as sugar, white flour, sweets, and sugary drinks) and processed fats and a strong reduction in micronutrients, fiber and omega-3 to omega-6 fatty acid ratios [17,19]. All the changes mentioned are correlated with an increase in inflammatory processes [20], so it is not surprising that all the dietary factors mentioned are also associated with an increase in gingival inflammation [21,22].

Based on this, important intervention studies were able to show that a focused intake of these substances was able to reduce gingival inflammation to a clinically relevant extent [23,24,25,26,27]. These studies focused on high-fiber dietary interventions, omega-3 fatty acids, micronutrient-rich plants, plant-based nitrates and avoidance of processed carbohydrates and processed fats—or from a dietary perspective focusing on the Paleolithic diet, mainly plant-based wholefoods or the Mediterranean diet. Many of these studies have established the inflammation-reducing effect of diet, even though the quantity of dental plaque had not changed or had even increased [23,24,25,26,27]. However, this correlation reveals a clear contradiction to the assumption stated in official classifications that dental plaque is the decisive etiologic factor for gingivitis [1]. If, on the other hand, the study situation was to confirm the etiological role of diet, this would have immense implications for the current dental care situation. Plaque control would then have to be given an effective, but only symptomatic role in the treatment and prevention of gum disease. Dental nutrition therapy, on the other hand, would have to be placed back at the center of prevention and treatment.

On this background, there is a high relevance to further investigate the etiological role of dental plaque and gingival inflammation. Even though there were single interesting studies showing an uncoupled relationship between plaque and gingival inflammation under different nutritional settings [23,24,25,26,27], there are a lack of summarized data on this relationship. Thus, the aim of the current study was to reanalyze the association of plaque and gingival inflammation in comparable nutritional studies.

## 2. Materials and Methods

This summarized secondary analysis was based on the data of four clinical trials (one cohort study [15], and three randomized clinical trials [23,26,27]), analyzing periodontal inflammation under changed dietary conditions.

### 2.1. Eligibility Criteria of Participants

While the participants of Baumgartner et al. did not have to fulfill any inclusion criteria, except their consent to live for four weeks under stone-age conditions, the other three studies had comparable inclusion and exclusion criteria.

#### 2.1.1. Inclusion Criteria

- Written informed consent to participate;
- Gingivitis (mean GI by Löe and Silness [28] ≥ 0.5)/Bleeding on probing [BOP] > 30%) [1]
- ≥20 teeth;
- Western diet conditions with a main criterion of a processed carbohydrate intake above 45% [29] or self-reported WD conditions with a daily intake of processed carbohydrates, sugar, and saturated fatty acids (Cena & Calder [30]);
- Age ≥ 18.

#### 2.1.2. Exclusion Criteria

- Periodontitis (CPITN by Ainamo et al. [31] two times ≥ 3 or 4);
- Smoking;
- Severe or life-threatening illnesses;
- Intake of antibiotics within 3/6 months before the start of or during the study period;
- Drugs influencing gingival inflammation or bleeding (e.g., anticoagulants, cortisone);
- Carbohydrate- or insulin-related diseases (e.g., diabetes);
- Pregnancy or breastfeeding.

Additionally, Bartha et al. excluded participants with a current intake of probiotics; dislike or intolerance of fish, milk, or milk products; allergies against fish, fruits, nuts; eating disorders (anorexia nervosa, bulimia, binge eating, or fasting); and primarily plant-based diet habits, including whole food vegetarian or whole food vegan diet styles, a low-carb diet, or a Mediterranean style diet. A detailed list of the inclusion and exclusion criteria is provided in Appendix A, Table A1.

### 2.2. Clinical Procedures

All studies comparably assessed the following clinical periodontal parameters.

Gingival inflammation was measured according to the GI by Löe and Silness (1963) [28], plaque values were measured according to the PI by Silness and Löe (1964) [32], and bleeding on probing (BOP) was measured full-mouth [33].

In each single study, the clinical assessment was performed by one examiner who was blinded to the group allocation, except the study of Baumgartner et al. [15], who did not include a control group. All studies included a baseline measurement and a final measurement after 4 weeks of intervention. The studies by Woelber et al. [26,27] and Bartha et al. [23] were performed in University clinical settings in Germany. The participants of the stone age project [15] lived for four weeks in Pfyn, Thurgau, Switzerland, but clinical assessments were performed before and after at the University of Zurich, Switzerland.

### 2.3. Nutritional Interventions

All nutritional Interventions Studied had common features:

- Absence or avoidance of processed carbohydrates (such as sugar, white flour, juice, soft-drinks);
- Avoidance of processed meat and limited intake of white meat;
- Focused intake of marine omega-3 fatty acids (such as fish or fish/algae oil);
- Focused intake of whole foods (such as whole grains, fruits, vegetables, nuts, and seeds), with a high amount of dietary fibers;
- Duration of the dietary intervention for 4 weeks.

Nonetheless, there were also some differences in detailed foods and nutrients. Baumgartner et al. [15] described a stone age diet without legumes. Because the food supply was not sufficient to provide the participants with a full diet over 4 weeks, the participants had also to seek supplemental food from nature, including berries, edible plants, and fish. The authors of [23] investigated a Mediterranean diet which—in contrast to a stone-age diet—included olive oil as a main fat source [34]. Woelber et al. [26,27] investigated a mainly plant-based whole-food diet. The dietary concept also included a focused intake of vitamin C-containing plants, vitamin D, nitrate-containing plants, and sources of antioxidants (such as berries, green tea, and curcumin). A detailed description of the applied diets, the diet monitoring procedures and related information are shown in Table 1.

### 2.4. Statistical Analysis

For descriptive analysis mean values and standard deviations were computed. Boxplots and scatter plots were used for graphical presentation. The changes ∆ were calculated as value at time 2—value at time 1 for all parameters. Linear regression models taking the clustering of the data due to several studies into account (option cluster within STATA) were applied to analyze the influence of plaque index changes on gingiva index changes as well as changes in values for bleeding on probing within each group. In order to obtain a numerical value for the ratio of GI to PI as well as BOP to PI—that is comparable between the studies—the quotient of GI and PI (respectively BOP and PI) was calculated for each time point and each patient. Linear mixed-models with study number and proband as the random effect were used to compare these ratios between the two time points within the pooled control and treatment groups, respectively. The results were verified by additionally adjusting for age and gender. The significance level was set to 0.05. For all analyses, the statistics program STATA (StataCorp LT, College Station, TX, USA, version 17.0) was used.

## 3. Results

**Figure 1:**
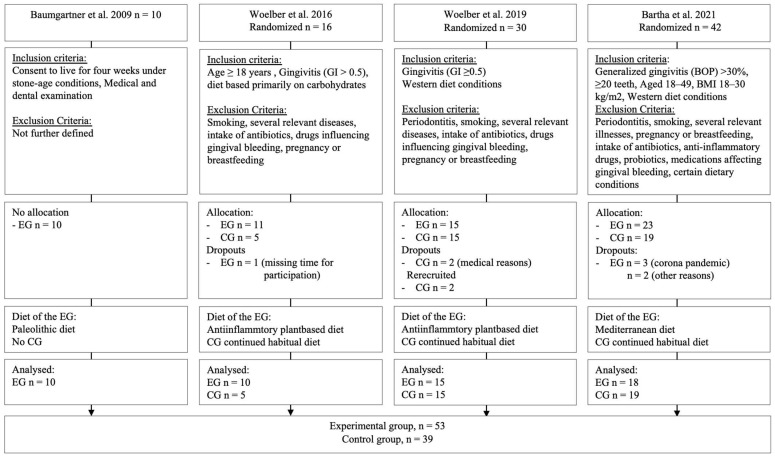
Flowchart regarding all included studies; EG = experimental group, CG = control group, BOP = bleeding on probing, GI = gingival index, BMI = body-mass-index [15,23,26,27].

Merging the study data of the four studies resulted in a total of 53 participants in the experimental group (dietary change) and 39 participants in the control group (continued Western diet). Dropouts occurred due to denying further participation due to the COVID-19 pandemic (n = 3 within the intervention group of [23]), or declining participation for other reasons (n = 2 within the intervention group of Bartha et al., 2021), due to medical reasons (phlebitis and sinusitis; n = 2 within the control group of [27]), and due to missing time for participation (n = 1 within the intervention group of [26]) (Figure 1). The distribution of age and sex within the studies and groups can be found in Appendix A (Table A2).

**Figure 2:**
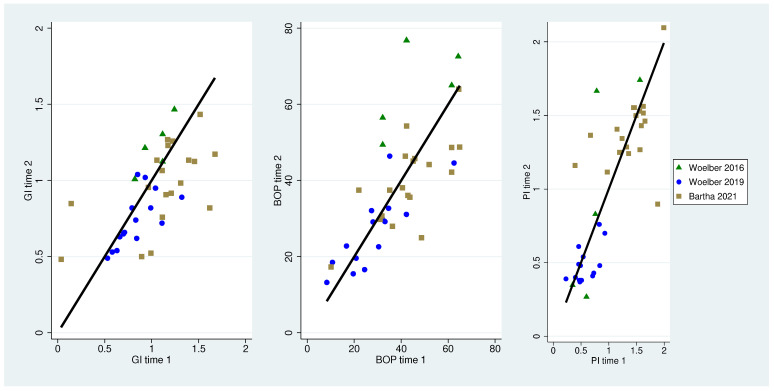
Association of clinical parameters (gingiva index, bleeding on probing and plaque index) between time 1 and time 2 for control groups. The black diagonal displays a change of zero, data left of the line are displaying higher values at time 2, and data right of the diagonal are displaying higher values at time 1 [23,26,27].

**Figure 3:**
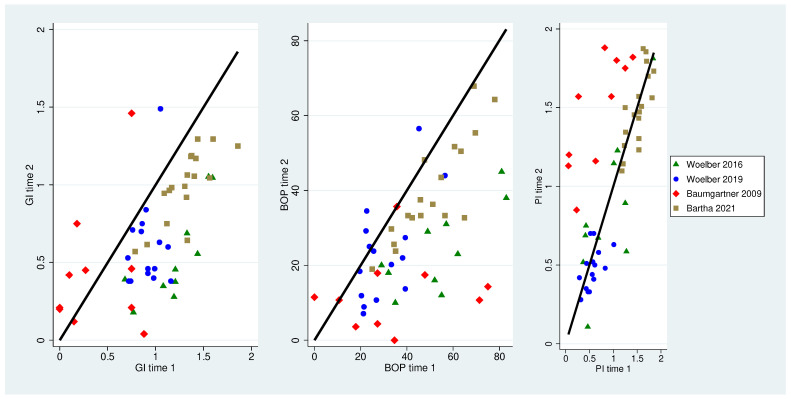
Association of clinical parameters (gingiva index, bleeding on probing and plaque index) between time 1 and time 2 for the test groups. The black diagonal displays a change of zero, data left of the line are displaying higher values at time 2, and data right of the diagonal are displaying higher values at time 1 [15,23,26,27].

In almost all patients in the treatment groups, the gingiva index and the bleeding on probing value could be reduced whereas the plaque index remained unchanged or showed higher values only in patients without tooth brushing [15] at the end of the study (Table 2 and Table 3). Values from timepoints 1 and 2 are displayed in Table 3. Visualization of the data (Figure 2 and Figure 3) showed that in the treatment groups, the magnitude of the bleeding values were located right from the zero-diagonal, displaying higher values at time 1. In contrast, the plaque values were beneath the diagonal except for the data of Baumgartner, with all data points left to the diagonal, displaying higher plaque values at time 2. Visualization of the control groups’ data displays the magnitude of data points near to the zero-diagonal.

### Relationship between Changes in Plaque and Bleeding Scores

**Figure 4:**
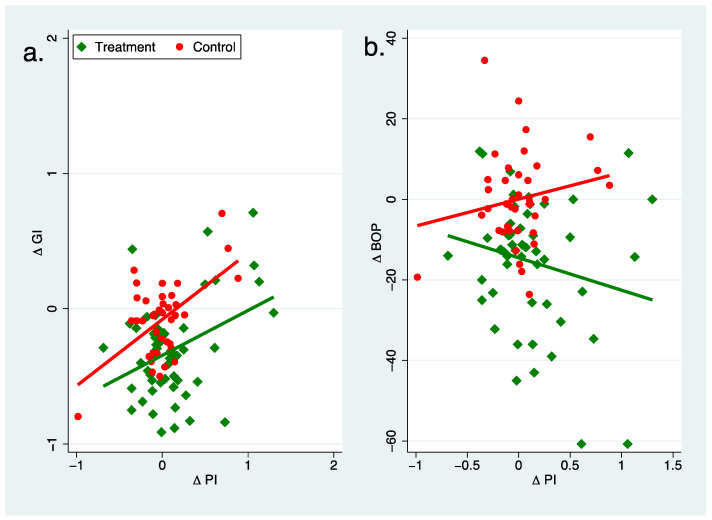
Association between change in plaque index and change in gingiva index (**a**) and bleeding on probing (**b**) for control and treatment group. Δ computed as value time 2 − time 1.

Visualizing the ratio between the changes of plaque and bleeding scores, positive values for the slopes of the regression lines (Figure 4) for the control group (GI: 0.49; BOP: 6.67) could be observed, while for the treatment group the slope for GI became smaller (0.33) and even negative for BOP (−8.03). When adjusting for gender and age, the following relationships were confirmed: control group GI: 0.48 BOP: 6.29; treatment group: GI: 0.33; BOP: −8.50. For both GI (*p* = 0.016) and BOP (*p* = 0.031), we observed significant group differences, which also remained stable after adjusting for age and sex (GI: *p* = 0.018; PI: *p* = 0.034).

**Figure 5:**
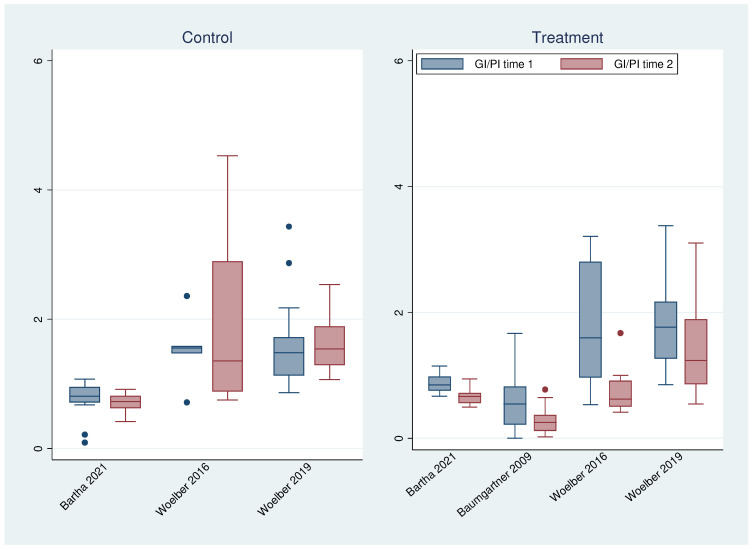
Boxplots of the ratio of GI and PI at time 1 and 2 for all studies and groups [15,23,26,27].

**Figure 6:**
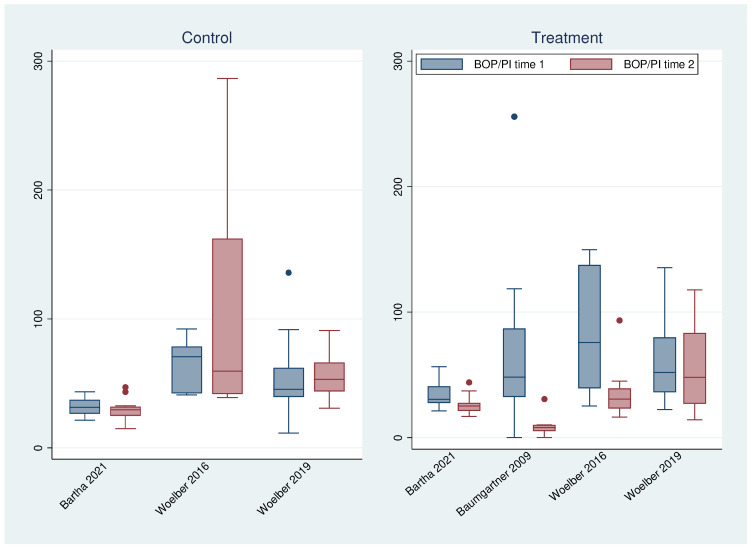
Boxplots of the ratio of BOP and PI at time 1 and 2 for all studies and groups [15,23,26,27].

To visualize this relationship in more detail, the quotient of GI (respectively BOP) and PI was calculated for each patient and each time point (Figure 5 and Figure 6). In all treatment groups—regardless of the study—these ratios were always significantly (common analysis over all treatment groups *p* < 0.001 for GI and BOP) lower at time point 2 than at time point 1, while no difference could be found for the control groups (GI *p* = 0.837; BOP *p* = 0.343). Since the plaque index did not really change over time (with the exception of the studies without tooth brushing), this was caused by changes in GI and, respectively, BOP.

## 4. Discussion

The aim of the study was to reanalyze the association of plaque and gingival inflammation in comparable nutritional studies in a secondary analysis. Results showed a significant and clinically relevant decrease in gingival inflammation, represented by decreased GI and BOP scores, in the absence of a significant change in plaque levels. With regard to GI, the nutritional intervention led both to a lower inflammatory level of GI/PI and a decreased ratio of GI/PI, indicating a kind of higher “plaque resistance”. With regard to BOP, the nutritional intervention led also to a lower level of BOP/PI, but furthermore to an inverted relationship between BOP and PI, indicating a beneficial effect of plaque accumulation (“healthy plaque”). Based on these results, there is a serious need to discuss whether plaque should really still be considered as an etiological factor or rather as a kind of “catalyst” of pro- or anti-inflammatory bases, such as those strongly induced by diet. This assumption is also consistent with current etiological considerations such as the ecological plaque hypothesis, the “keystone pathogen” hypothesis, and the integrated caries and periodontal inflammation hypothesis [9,10,11]. The results have to be discussed in several directions.

Regarding the mode of action, it can be assumed that the nutritional intervention had an impact on both plaque quality and the inflammatory response of the periodontal tissues. Prior studies have shown that processed carbohydrates and saturated fatty acids can have a pro-inflammatory effect on gingival tissues by the production of short-chain carboxylic acids [35]. This would be in line with the integrated hypothesis of dental caries and periodontal diseases formulated by Nyvad and Takahashi [11], describing fermentable carbohydrates (such as free sugar) as both pro-inflammatory and cariogenic.

Looking at the interrelationships between diet and the corresponding microbiome, it is known that dietary changes can have an early impact, especially on the supragingival microbiota [36]. The subgingival microbiota in the study of Woelber et al., 2019 did not show any significant changes due to the nutritional intervention [27]; however, the analysis of the supragingival plaque in the Woelber et al., 2016-study showed a reduction in the total counts of *Streptococcus mitis* group, *Granulicatella adiacens*, *Actinomyces* spp., and *Fusobacterium* spp. in the healthy diet group [34]. While there were no microbiota-associated data available in the study by Bartha et al. [23], Baumgartner et al., (2009) [15] identified a significant increase in the bacterial counts for 24 of 74 species in the subgingival plaque samples, including *Actinomyces odontolyticus*, *A. vaginae*, *B. ureolyticus*, *Eikenella corrodens*, *L. acidophilus*, *Capnocytophaga ochracea*, *Dialister* sp., *Escherichia coli*, *Fusobacterium nucleatum naviforme*, *Gardnerella vaginalis*, *Haemophilus influenzae*, *H. pylori*, *L. crispatus*, *Lactobacillus jensenii*, *N. mucosa*, *Peptoniphilus* sp., *Porphyromonas endodontalis*, *Prevotella disiens*, *Prevotella mirabilis*, *Staphylococcus aureus* (two strains), *Streptococcus agalactiae*, *S. anginosus*, and *S. mitis*. However, the study authors judged the found strains as not classically associated with tooth decay or periodontitis [15]. Another mode of action can be assumed in a diet-based host-modulatory effect. All common features of the study diets (such a restriction of processed carbohydrates and omega-6 fatty acids, and a focused intake of fibers, omega-3 fatty acids, plant nitrates, and micronutrients) have been described with anti-inflammatory or inflammation-resolving effects on periodontal tissues [21,37,38]. Although the Woelber et al. 2019 study could not find any reduction in serum inflammatory parameters (assessed by Interleukin 6, Tumor necrosis factor alpha, and C-reactive protein), three studies [15,23,27] observed anthropometric effects in the form of significant weight loss in the participants of the experimental groups (of about 1.5 kg during four weeks). This latter result is in line with a study showing a significant excess calorie intake under ultra-processed-diet environments [39].

With regard to the effect size, the nutritional interventions can be compared to meta-results from oral hygiene-related studies [40,41,42]. Compared to the found reduction for GI of −0.34 points, Berchier et al. found a reduction of −0.08 points for the additional use of flossing to manual tooth brushing [43]. Comparing BOP and GI with regard to the use of interdental brushes, a meta-analysis by Slot et al. found reductions in BOP within 44–53% for the use of interdental brushes additionally to tooth brushing, depending on the study and the baseline values [44,45]. Accordingly, the found effects of the nutritional interventions seem to be more effective than additional flossing but less effective than interdental brushes. However, it has to be kept in mind that both approaches (plaque reduction and nutritional interventions) seem to have different modes of action and thus easily can be combined for the best of both worlds.

While the found effect of diet-induced “plaque resistance” is still in line with the concept of “plaque-induced” gingivitis (although with a lesser relationship), the found results regarding the inverse relationship between plaque and BOP would reject the idea that gingival inflammation is “induced” by a plaque biofilm. It would even, on the contrary, attribute a gingivitis-protective effect to plaque, which we would formulate as the “healthy plaque hypothesis”. This hypothesis is based on the idea that Homo sapiens has changed its environment and living conditions in the course of human history towards today’s Anthropocene in such a way that plaque is not protective anymore but pathogenic [16,17,18]. Looking at the difference of living conditions of wild-living animals (who are commonly not depended on cultural oral hygiene) and prehistoric ancestors, it seems obvious that the Anthropocene shows new risk factors such as smoking, ultra-processed foods, and continuous stress.

Furthermore, three of the analyzed studies [15,23,27] found additional beneficial effects on overall health in form of weight loss, which indicates beneficial “downstream” effects for both oral and overall health. Accordingly, the investigated nutritional interventions are in line with the common risk factor approach focusing on effective interventions addressing several health problems with one intervention [46].

Besides the several common features of the investigated studies, the main limitations of the current investigation can be seen in the differences between them. This applies not only to the time of intervention (the Baumgartner study [15] was performed about seven years before the Woelber et al., 2016 study [26]), but also to the investigated diets and further influencing variables. Presumably, most relevantly, the participants of the Baumgartner study were living under total different circumstances compared to the other three studies, including more physical activity and presumably less stress. Both factors were shown to have a beneficial influence on periodontal tissues [47,48]. On the other hand, one of the most important risk factors, smoking, was controlled in all studies and all studies had a comparable duration. A further limitation can be seen in the missing calibration between the study examiners. Both studies by Woelber et al. were performed with a pressure-sensitive periodontal probe. The otherwise missing calibration between the study dentists was statistically taken into account as part of the confounders. Nevertheless, future studies with a comparable number of participants with calibrated examiners would be beneficial.

## 5. Conclusions

Diet seems to be one determining factor on how the gingival tissues react towards dental plaque accumulation, which should be considered in classifications and etiological hypotheses. Current Western dietary conditions seem to have a profound pro-inflammatory effect on periodontal tissues, while wholefood diets with lots of fiber and micronutrients, as well as omega-3 fatty acids, are associated with a lower level of inflammation in the periodontium. The effect size of the diets studied has both clinical and public health relevance and emphasizes the central role of nutritional advice and therapy in dental practice. Further clinically controlled studies are necessary to investigate these effects and underlying mechanisms.

## Figures and Tables

**Table 1:** Detailed description of the applied diets and relating methods of all four studies (GI = Gingival index by Löe and Silness (1963) [28]; PI = Plaque index by Silness and Löe (1964) [32]; BOP = relative number of sites with bleeding on probing).

	Baumgartner et al., 2009 [15]	Woelber et al., 2016 [26]	Woelber et al., 2019 [27]	Bartha et al., 2021 [23]
Applied diet concept	“basic supply of whole grains of barley, wheat, spelt (“einkorn”, “emmer” = local ancient agricultural wheat), some salt, herbs, honey, milk, and meat from domestic animals (goats and hens)” Participants were “forced to seek supplemental food from nature, including berries, edible plants, and fish without nets” No access to refined sugars or modern kitchen utensils	“Reduction of the intake of carbohydrates as far as possible to a level <130 g/d, including a restriction of fructose, disaccharides, sweetened beverages and meals, flour containing foods, rice and potatoes as far as possible. No restrictions regarding fruits and vegetables (polysaccharides) as long as the total amount of carbohydrates was considered. Daily intake of Omega-3 fatty acids, a restriction in the amount of trans-fatty acids as far as possible A reduction in Omega-6 fatty acids as far as possible Daily intake of a source of vitamin C Daily intake of a source of vitamin D (15 min unprotected in the sun, supplementation with 500 international units (12.5 μg) Daily intake of antioxidants (such as a handful of berries, cup of green tea, coffee, etc.) Daily intake of fiber (vegetables and fruits).”	See [26] with the following modifications: “Reduction of industrial animal proteins (like industrial dairy and meat products) as far as possible and favouring of plant proteins (like legumes, nuts, etc.) Daily intake of nitrate-containing plants (such as a portion of spin ach, beet root, or rocket) Daily intake of a source of vitamin D either by exposing the body 15 min unprotected in the sun or supplementation with 1000 international units (25 μg). In case of lower baseline serological values, (≤30 ng/mL) individually higher values were administered”.	According to [34] A minimum of one to two servings of whole grains per meal, such as bread, pasta, rice, couscous, and other similar foods. At least two servings of vegetables per meal. Two to three servings of fruit per meal. Daily 1.5 to 2 L of water. Moderate consumption of dairy products. Olive oil as main source of fats Daily intake of olives, nuts, and seeds. Regular inclusion of spices, herbs, garlic, and onions. Optional moderate intake of wine during meals, (one glass for women and two for men per day) Weekly two portions of seafood and shellfish, poultry, Eggs (two to four servings) Less than two servings of red and processed meats Legumes at more than two servings per week potatoes at one to two servings per week. A recommendation to minimize sugar and sweets, keeping them occasional.
Duration of Intervention	Four weeks	Four weeks	Four weeks	Four weeks + first two weeks nutritional transition
Intervention method	“Participants lived in an environment, developed by anthropologists to be as similar as possible to what had been identified in archeologic findings in early Stone Age or between 4000 and 3500 BC”	“Dietary recommendations were delivered verbally (30 min) and by handing out an information brochure containing an additional list of restricted and recommended foods and meals. After one week, participants were asked about their experiences and possible problems. When more information was needed, participants had the chance to contact two of the authors at any time during the study”	“Detailed verbal introduction into the AID protocol for 30 min by one of two nutritional dentists specialized in nutritional medicine. The participants were informed to contact the study center for any help regarding the dietary recommendations.”	Participants had to participate in at least three of four Mediterranean training sessions, each lasting 45–60 min, supplemented with homework tasks and two information brochures. The sessions were provided by a dietician and a dentist specialized in clinical nutrition. The participants were able to contact the study center for any help regarding the Mediterranean diet.
Clinical parameters and measurements	BOP, PI, GI (GI-data provided from corresponding author)	BOP, GI, PI	BOP, GI, PI	BOP, GI, PI
Diet adherence recording	Participants lived for 4 weeks full time in a stone age environment, accompanied by the swiss television.	“Participants filled out a daily food diary throughout the study duration”	“Participants filled out a 24 h dietary diary for 1 week at the second, fifth and eighth week”	“Participants completed the German Health Interview and Examination Survey for Adults Food Frequency Questionnaire (DEGS-FFQ) (Robert-Koch-Institute, Berlin, Germany) and the Mediterranean Diet Adherence Screener (MEDAS)”
Oral hygiene restrictions	Only the use of twigs and any other natural material was allowed.	Tooth brushing without the use of interdental brushes or dental floss.	Tooth brushing without the use of interdental brushes or dental floss.	Tooth brushing without the use of interdental brushes or dental floss.

**Table 2:** Mean values (±standard deviation) and number of patients (n) regarding the changes of gingival index, plaque index and bleeding on probing of the treatment (T) and control groups (C) of all included studies.

Study	Group	n	ΔGingiva Index	ΔPlaque Index	ΔBleeding on Probing
Bartha et al., 2021 [23]	C	19	−0.14 (±0.35)	0.02 (±0.36)	−3.47 (±10.10)
	T	18	−0.31 (±0.16)	−0.02 (±0.16)	−11.07 (±7.60)
Baumgartner et al., 2009 [15]	T	10	0.05 (±0.48)	0.80 (±0.31)	−22.15 (±24.80)
Woelber et al., 2016 [26]	C	5	0.18 (±0.10)	0.16 (±0.45)	17.60 (±12.42)
	T	10	−0.67 (±0.19)	−0.04 (±0.32)	−29.30 (±12.38)
Woelber et al., 2019 [27]	C	15	−0.09 (±0.16)	−0.09 (±0.16)	−1.47 (±7.78)
	T	15	−0.31 (±0.26)	−0.08 (±0.16)	−6.80 (±11.01)
Overall	C	39	−0.08 (±0.28)	−0.01 (±0.31)	−0.003 (±11.60)
	T	53	−0.31 (±0.36)	0.11 (±0.40)	−15.39 (±16.07)

**Table 3:** Number of patients (n) and mean values (±standard deviation) of clinical parameter in the control (C) and treatment (T) group of the studies examined.

Study	Group	n	Gingiva Index		Plaque Index		Bleeding on Probing	
			Time 1	Time 2	Time 1	Time 2	Time 1	Time 2
Bartha et al., 2021 [23]	C	19	1.12 (±0.42)	0.97 (±0.27)	1.38 (±0.39)	1.40 (±0.24)	43.22 (±14.25)	39.74 (±11.01)
	T	18	1.31 (±0.25)	1.00 (±0.23)	1.51 (±0.21)	1.49 (±0.24)	51.00 (±14.65)	39.93 (±13.74)
Baumgartner et al., 2009 [15]	T	10	0.38 (±0.35)	0.43 (±0.42)	0.68 (±0.50)	1.47 (±0.36)	34.77 (±24.30)	12.62 (±10.02)
Woelber et al., 2016 [26]	C	5	1.04 (±0.17)	1.22 (±0.17)	0.81 (±0.46)	0.97 (±0.70)	46.46 (±15.61)	64.06 (±11.27)
	T	10	1.20 (±0.30)	0.54 (±0.30)	0.88 (±0.49)	0.84 (±0.47)	53.50 (±18.68)	24.20 (±11.39)
Woelber et al., 2019 [27]	C	15	0.83 (±0.22)	0.74 (±0.18)	0.57 (±0.19)	0.48 (±0.12)	28.39 (±13.31)	26.92 (±9.90)
	T	15	0.92 (±0.14)	0.61 (±0.29)	0.56 (±0.18)	0.48 (±0.13)	30.35 (±11.07)	23.55 (±13.62)
Overall	C	39	1.00 (±0.35)	0.92 (±0.28)	0.99 (±0.51)	0.99 (±0.52)	37.93 (±15.71)	37.93 (±15.69)
	T	53	1.00 (±0.42)	0.69 (±0.37)	0.97 (±0.53)	1.08 (±0.54)	42.57 (±19.19)	27.17 (±15.94)

## Data Availability

Data will be available from the corresponding author on reasonable request.

## Appendix A

**Table A1:** Detailed inclusion and exclusion criteria as given within each study.

	Baumgartner et al., 2009 [15]	Woelber et al., 2016 [26]	Woelber et al., 2019 [27]	Bartha et al., 2021 [23]
Inclusion criteria	Consent to live for four weeks under stone-age conditions Medical and dental examination before the study and 4 weeks after. No further details provided	Age ≥ 18 years Presence of gingivitis (GI > 0.5) A diet based primarily on carbohydrates	Gingivitis (mean GI by Löe and Silness (1963) ≥0.5) Western diet conditions with a main criterion of a processed carbohydrateintake above 45%, age ≥ 18	Generalized gingivitis (bleeding on probing [BOP] > 30%) ≥20 teeth Aged 18–49 with a body mass index (BMI) of 18–30 kg/m^2^ Self-reported WD conditions assessed by verbal anamnesis and defined as a daily intake of processed carbohydrates, sugar, and saturated fatty acids
Exclusion criteria		Smoking Infectious or life-threatening diseases Intake of antibiotics within 3 months before the start of or during the study period. Drugs influencing gingival inflammation or bleeding (e.g., anticoagulants, cortisone) Carbohydrate- or insulin-related diseases (e.g., diabetes) Pregnancy or breastfeeding	Periodontitis (CPITN two times ≥ 3 or 4) Smoking Severe or life-threatening illnesses Intake of antibiotics within 6 months before the start of or during the study period Drugs influencing gingival inflammation or bleeding (e.g., anticoagulants, cortisone) Carbohydrate- or insulin-related diseases (e.g., diabetes) Pregnancy or breastfeeding.	periodontitis (CPITN two times > 2); Smoking Severe illnesses (e.g., HIV, chronic hepatitis, cancer, illnesses of the salivary glands or gastrointestinal tract, or diabetes mellitus); Pregnancy or breastfeeding Intake of antibiotics 6 months prior or during the study Intake of anti-inflammatory drugs Intake of medication affecting gingival bleeding Intake of probiotics Dislike or intolerance of fish, milk, or milk products Allergic to fish, fruits, and nuts Eating disorders (anorexia nervosa, bulimia, binge eating, or fasting) Primarily plant-based diet habits, including whole foods vegetarian or whole foods vegan diet styles, low carb diet, Mediterranean style diet.

**Table A2:** Distribution of age (mean ± SD) and gender (n, %) in the different studies and groups.

Study	Group	n	Age in Years At Baseline	Female n (%)
Bartha et al., 2021 [23]	C	19	29.4 (±7.3)	12 (63.2)
	T	18	33.0 (±9.1)	8 (44.4)
Baumgartner et al., 2009 [15]	T	10	28.1 (±15.6)	5 (50.0)
Woelber et al., 2016 [26]	C	5	34.0 (±16.5)	3 (30.0)
	T	10	34.4 (±14.1)	6 (60.0)
Woelber et al., 2019 [27]	C	15	33.7 (±13.1)	7 (46.7)
	T	15	27.2 (±4.7)	9 (60.0)
Overall	C	39	31.6 (±11.1)	22 (56.4)
	T	53	30.7 (±10.9)	28 (52.8)
